# One minute of light‐intensity stair‐stepping decreases postprandial glycaemia in the evening in non‐diabetic adults: A randomized controlled trial

**DOI:** 10.1113/EP092274

**Published:** 2024-12-20

**Authors:** Austin Morales, William Wong, Jeff Moore, Jochen Kressler

**Affiliations:** ^1^ School of Exercise and Nutritional Sciences San Diego State University San Diego CA USA

**Keywords:** cardiovascular, diabetes, evening, exercise, glycaemia, metabolism, postprandial, stair‐stepping

## Abstract

Prior studies have investigated the efficacy of a single 1 min bout of stair‐stepping on reducing postprandial blood glucose (BG) in the morning, but none have investigated this effect in the evening when glycaemic responses are larger due to circadian regulation and β‐cell responsiveness. This work investigated the efficacy of a 1 min bout of self‐selected, low‐intensity stair‐stepping performed in the evening on reducing the change from baseline to the 60 min time point postprandial BG. Thirty people (43% male, 29 (10) years) participated in a randomized crossover‐controlled trial. Participants completed two separate evening trials following an oral glucose tolerance test (OGTT) (75 g of dextrose dissolved in water): (a) 0 min of stair‐stepping seated control condition, and (b) 1 min of stair‐stepping condition. One minute of stair‐stepping attenuated the change from baseline to 60 min postprandial BG versus control condition (2.5 (2.8) vs. 4.3 (2.3) mmol/L (*P* < 0.001). Area under the curve (AUC) and incremental area under the curve (iAUC) were lower for the 1 min condition versus control (mean difference = −0.4,95% CI: 0.1–0.8 (*P* = 0.023) and (mean difference = −0.6, 95% CI 0.1–1.1 mmol/L^−1^ min^−1^ (*P* = 0.043), respectively. The modified BORG rate of perceived exertion scale showed participants perceived the exercise as light intensity (1.9 (1.1)). A single, 1 min bout of low‐intensity stair stepping at a self‐selected pace reduced evening postprandial BG levels following an OGTT in young non‐diabetic adults.

## Abstract (highlights)

- **What is the central question of this study?**
  What is the efficacy of a 1 min bout of self‐selected, low‐intensity stair‐stepping performed in the evening on reducing the change from baseline to the 60 min time point postprandial blood glucose?
- **What is the main finding and its importance?**
  A single 1 min bout of low‐intensity stair stepping reduced evening postprandial blood glucose levels in young non‐diabetic adults.

## 1 INTRODUCTION

The prevalence of type 2 diabetes mellitus is increasing in the USA, with 11.6% of people being classified as diabetic and 38% classified as pre‐diabetic, as of 2021 (Centers for Disease Control & Prevention, 2021.). Factors such as increased adiposity and lack of physical activity perpetuate the prevalence and severity of type 2 diabetes mellitus. The postprandial phase is particularly important as increased postprandial blood glucose (BG) is associated with higher risk of diabetes, cardiovascular disease and death (Hiyoshi et al., 2017; Kahn, 2003; Temelkova‐Kurktschiev et al., 2000). Further, the risk of disease is increased for people with diabetes, as well as non‐diabetics, in response to elevated postprandial BG (Levitan et al., 2004).

Exercise is an effective strategy to help reduce postprandial BG. Chronic physical activity can increase GLUT4 expression, insulin sensitivity, and allow for GLUT4 translocation independent of insulin, but most Americans are not meeting general recommendations for physical activity (Draicchio et al., 2022; Richter & Hargreaves, 2013). Exercise options that are both convenient and accessible may help the general public lower postprandial BG. Stair‐stepping exercise is a promising option as it is inexpensive, and staircases are commonly accessible for many people. It is estimated that 47% of homes nationwide require stairs to enter the home (Office of Policy Development and Research, n.d.). Workplaces and commercial buildings also have staircases, increasing stair‐stepping's accessibility. Additionally, stair‐stepping appears to be more effective than alternatives such as cycling or steady‐state walking in regards to improving glycaemia over short durations (Bellini et al., 2021; Takaishi & Hayashi, 2017; Takaishi et al., 2012). Stair‐stepping has previously been investigated regarding its postprandial BG lowering efficacy. Single bouts of 1, 3 and 10 min at a self‐prescribed comfortable pace were effective in lowering postprandial BG in response to an oral glucose tolerance test (OGTT) and a mixed meal and effective regardless of sex or cardiorespiratory fitness level (Bartholomae et al., 2018; Moore et al., 2022; Moore, Bartholomae et al., 2020; Moore, Salmons et al., 2020).

While the previous stair‐stepping studies have shown reductions in postprandial BG, all of these studies were conducted in the morning following an overnight fast. Glucose control changes throughout the day with higher postprandial BG responses in the evening than in the morning (Draicchio et al., 2022; Jarrett et al., 1972; Poggiogalle et al., 2018). Circadian rhythms influence glucose control with a diurnal rhythm. The diurnal effect can be substantial to the point where those with normal glucose control in the morning are metabolically equivalent to individuals with pre‐diabetes in the evening (Poggiogalle et al., 2018). β‐Cell responsiveness also contributes to the elevated BG levels observed in the evening. Rates of insulin secretion are higher in the evening and lower in the morning in response to an OGTT (Fujimoto et al., 2022).

Although exercise of sufficient duration and intensity can ameliorate late‐day glucose response, no study has investigated the effects of short, single bouts of stair‐stepping exercise on postprandial glucose in the evening (Colberg et al., 2009). The purpose of this study is to investigate the effects of a 1 min bout of stair‐stepping on evening postprandial glycaemia, following ingestion of 75 g of dextrose. We hypothesized that stair‐stepping would reduce the change from baseline to 60 min postprandial glycaemia compared to a seated control condition.

## 2 METHODS

### 2.1 Participants

#### 2.1.1 Ethical approval

All participants provided written informed consent prior to beginning the study. The study was approved by the Institutional Review Board (IRB) at San Diego State University (SDSU). (The SDSU IRB is accountable for the review of human‐subject research to ensure it meets applicable federal regulatory requirements found at 45 CFR 46, 21 CFR 50, and 56, all state and local laws, institutional policies, and the ethical principles within the Ethical Principles and Guidelines for the Protection of Human Subjects of Research also known as the Belmont Report.)

#### 2.1.2 Screening

All participants were assessed for cardiovascular risk via the PAR‐Q+ (Physical Activity Recall Questionnaire). Thirty healthy adult participants (43% male, 29 (10) years) were recruited and completed the study. All participants were self‐reported as healthy. Exclusion criteria consisted of women who were pregnant or planning to become pregnant. Participants’ prior exercise history was not assessed, but participants were instructed to refrain from physical activity the day of trials. Participants were instructed to have similar dietary intakes on the days of the trials. Participants were asked to maintain their diet and lifestyle habits throughout their participation. All data is expressed as a means (SD) (Table 1).

**TABLE 1:** Baseline participant characteristics.

	Age (years)	Resting SBP/DBP (mmHg)	Resting heart rate (bpm)	Average BMI (kg/m^2^)	Baseline BG (mmol/L)
All (*n* = 30)	29 (10)	122 (13)**/**77 (8)	70 (12)	25 (4)	5.3 (0.5)
Male (*n* = 13)	32 (2)	128 (13)**/**76 (7)	66 (9)	25 (2)	5.4 (0.4)
Female (*n* = 17)	27 (9)	117 (10)**/**78 (9)	72 (13)	24 (4)	5.3 (0.6)

Abbreviation: BG, blood glucose; BMI, body mass index; DBP, diastolic blood pressure; SBP, systolic blood pressure.

### 2.2 Study design

We utilized a randomized crossover design with a total of two visits. All visits were conducted in the evening, no earlier than 17.00 h, and always at the same time (within 1 h) for both conditions. Both visits were conducted within 7 days of each other. Participants were fasted for at least 5 h prior to each trial. Upon arrival, baseline blood pressure, heart rate and fasting blood glucose were taken. After completion of baseline measurements, an OGTT was administered for each condition. The OGTT consisted of 75 g of dextrose powder dissolved in water. Participants completed an OGTT under two conditions: (a) a seated OGTT with no exercise intervention, and (b) an OGTT with a stair‐stepping exercise bout 57 min after consumption of dextrose solution. Both conditions were 120 min in duration, and the order of the trials was randomized. The exercise intervention was 1 min of stair stepping, up and down 32 steps in a stairway (step height 18 cm) at a self‐selected, comfortable pace. During the exercise intervention, participants wore a heart rate monitor strap.

### 2.3 Measurements

Heart rate and blood pressure were measured at the beginning of each condition. Heart rate was measured during the 1 min of exercise in order to determine peak heart rate.

BG measurements were taken using capillary samples from a fingerstick and a glucometer (Contour Next, Basel, Switerland). The measurements were taken at baseline, 30, 60, 70, 80, 90 and 120 min. Multiple glucose measurements were taken until two within 15 mg/dl were attained and they were subsequently averaged in accordance with ISO reproducibility standards (Freckmann et al., 2015). The modified BORG rating of perceived exertion (RPE) scale of 1–10 was used for participants to describe the peak level of intensity they experienced during the exercise trial.

### 2.4 Statistical analysis

Statistical analyses were performed using GraphPad Prism (GraphPad Software, Boston, MA, USA). Max heart rate was estimated using 220 minus age. Normality of data was assessed using the Shapiro–Wilk test. Outliers identified with the ROUT test were removed. Change from baseline to 60 min postprandial BG was calculated by subtracting baseline values from the 60 min time point. Area under the curve (AUC) for BG was calculated using the trapezoidal rule. Incremental AUC (iAUC) was calculated by subtracting baseline blood glucose levels from AUC. BG over time was analysed using a 2 (condition) by 7 (time) RMANOVA. Significant main effects and interactions were followed up with post‐hoc comparisons. Student's paired *t*‐test, if normally distributed, or Wilcoxon's matched‐pairs signed rank test, if non‐normally distributed, was used to analyse simple effect differences between conditions for change from baseline to peak AUC, and iAUC. Significance was set *a priori* at α = 0.05.

## 3 RESULTS

**FIGURE 1:**
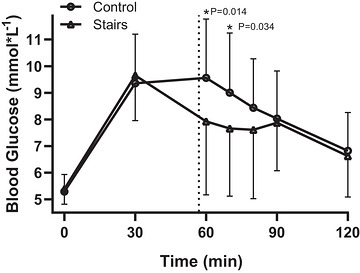
BG response over time for control and stair‐stepping conditions. Dotted line represents the onset of exercise.

For glucose over time (Figure 1) there was a significant interaction between time and group (*F* = 4.758, df = 6, *P* ≤ 0.001). Simple effect analysis showed no effect of condition before stair stepping (mean difference = −0.3 (0.4) [95% CI −1.1 to −0.4] mmol/L, *P* = 0.445), but significantly lower values compared to control after stair stepping at the 60 min (mean difference = −1.6 (0.7) [95% CI −2.9 to −0.3] mmol/L, *P* ≤ 0.014) and 70 min (mean difference = −1.3 (0.6) [95% CI −2.6 to −0.1] mmol/L, *P* = 0.034) time points, respectively. Smaller differences persisted at 80 min (mean difference = −0.8 (0.6) [95% CI −2.0 to 0.3] mmol/L, *P* = 0.159) but virtually no difference remained at 90 min (mean difference = −0.2 (0.4) [95% CI −0.8 to 0.6] mmol/L, *P* = 0.747) or 120 min (mean difference = −0.2 (0.4) [95% CI −0.9 to 0.6] mmol/L, *P* = 0.634).

**FIGURE 2:**
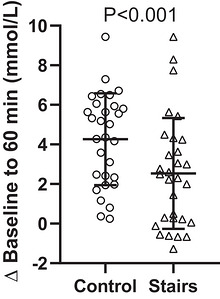
Mean difference in BG concentration from baseline to 60 min (60 min − baseline).

The change from baseline to 60 min postprandial BG (Figure 2) was lower during the stair‐stepping than the control condition (mean difference = −1.7 (2.3) [95% CI −2.6 to −0.9] mmol/L, *P* ≤ 0.001).

**FIGURE 3:**
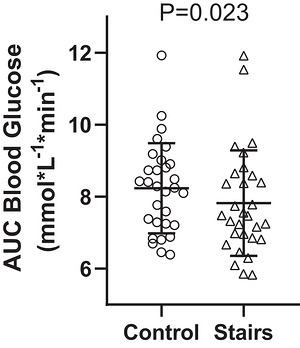
Mean (SD) area under the curve (AUC) values for control and stair‐stepping conditions.

**FIGURE 4:**
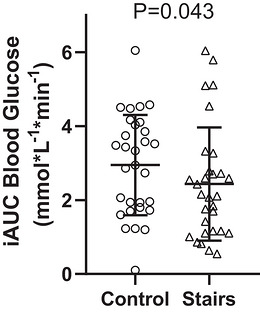
Mean (SD) incremental area under the curve (iAUC) values for control and stair‐stepping conditions.

**FIGURE 5:**
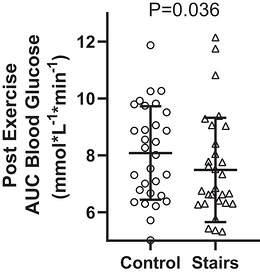
Mean (SD) area under the curve (AUC) values for control and stair‐stepping conditions, beginning at 60 min.

**FIGURE 6:**
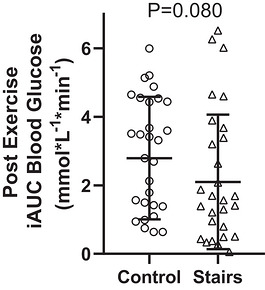
Mean (SD) incremental area under the curve (iAUC) values for control and stair‐stepping conditions, beginning at 60 min.

Stair‐stepping reduced both AUC (mean difference = −0.4 (0.9) [95% CI −0.8 to −0.1] mmol/L^−1^ min^−1^, *P* = 0.023, Figure 3) and iAUC (mean difference = −0.6 (1.3) [95% CI −1.1 to −0.1] mmol/L^−1^ min^−1^, *P* = 0.043, Figure 4) compared to the control condition. When considering the onset of exercise (57 min), stair stepping reduced the AUC (median = −0.6 [95% CI −1.1 to −0.1] mmol/L^−1^ min^−1^, *P* = 0.036, Figure 5) but not iAUC (median = −0.6 [95% CI −1.0 to −0.0] mmol/L^−1^ min^−1^, *P* = 0.080, Figure 6). Both heart rate (116 (14) bpm or 61% of estimated maximal heart rate) and RPE (1.9 (1.1)) indicated stair‐stepping was light to moderate intensity (Garber et al., 2011).

## 4 DISCUSSION

This study was the first to examine a single, 1 min bout of stair stepping on late day postprandial glucose responses. We saw a substantial 40% reduction in the difference in baseline to 60 min postprandial BG favouring the stair‐stepping condition. Previous studies conducted in the morning after an overnight fast found reductions in postprandial blood glucose with short single bouts of stair stepping, but of a lower magnitude (Moore, Bartholomae et al., 2020; Moore, Salmons et al., 2020; Moore et al., 2022). Exaggerated late day postprandial glucose responses are likely contributing to the relatively large effects observed in this study. Alterations in glucose control are seen throughout the day and postprandial responses are more pronounced in the evening (Fujimoto et al., 2022; Jarrett et al., 1972; Poggiogalle et al., 2018). One possible explanation for this occurrence is the diurnal rhythm exhibited in glucose control resulting in better glucose utilization in the morning than in the evening. Pancreatic β‐cell responsiveness and insulin sensitivity have been shown to be higher in the morning while insulin secretion rate and total insulin secretion in response to a meal are higher later in the day (Poggiogalle et al., 2018). Further, insulin resistance seen in the evening may in part be due to the rhythmic pattern of genes in skeletal muscles (Poggiogalle et al., 2018). Our study demonstrated excursions in glucose control in this evening study similar to or greater than other late day and evening reports (Colberg et al., 2009; Li et al., 2018). It is possible that the exaggerated glucose response seen in the evening helps explain the larger reductions seen in the current study compared to our previous work after overnight fasts as larger concentrations of blood glucose were available for increased disposal after stair stepping (Moore, Bartholomae et al., 2020; Moore, Salmons et al., 2020; Moore et al., 2022). In addition, higher levels of insulin in the evening may render stair stepping more efficacious due to the insulin sensitizing effects of exercise (Jarrett et al., 1972). Compared to previous studies, the level and duration of glucose reductions were equal or larger, extending for about 20 min, while previous research saw the effect limited to 15 min in the morning following exercise bouts of 1, 3 and 10 min (Moore, Bartholomae et al., 2020; Moore, Salmons et al., 2020; Moore et al., 2022). Previous evening studies have shown consistent results with our study, but with longer (20–60 min) exercise protocols performed (Colberg et al., 2009; Kim et al., 2022; Li et al., 2018).

Other exercise interventions have investigated the effects of exercise on postprandial blood glucose concentration. Factors such as exercise modality, duration of exercise and timing of exercise intervention have been looked at (Bellini et al., 2021; Moholdt et al., 2021). One important factor observed from prior research was the timing of exercise bouts (Engeroff et al., 2023). Oberlin et al. (2014) showed that performing aerobic exercising prior to breakfast, and at other points during the day, at 60–75% of max heart rate showed significant postprandial BG reductions following a 60 min exercise intervention. Conversely, most studies seemed to point to beginning exercise 15 min after a meal to have the most influential implications for postprandial BG (Bellini et al., 2021). We targeted our intervention to coincide as closely as possible with the anticipated peak postprandial BG response, which was 60 min after ingestion of the glucose solution. Whether earlier timing would have increased the magnitude and or duration remains to be investigated.

It is yet undetermined why such a short, light‐intensity bout of stair stepping has such a substantial effect on postprandial glycaemia. It is important to note the specific intervention of stair stepping appears to be particularly effective for reducing postprandial BG, as other studies have noted mixed results with flat walking and greater efficacy when compared directly to longer duration (6–60 min) of walking or cycling (Bellini et al., 2021; Diekmann et al., 2019; Oberlin et al., 2014; Takaishi et al., 2012). A potential mechanism for this is the mechanical changes stair stepping induces. Specifically, the protein RAC1 within the extracellular matrix may contribute to GLUT4 translocation during expansion or stretch of the muscle (Draicchio et al., 2022). RAC1 seems to be important in helping facilitate the connection between integrin, talin, and vinculin, which support glucose uptake into the muscle cell (Draicchio et al., 2022). Varying levels of insulin resistance may have also contributed to our findings. Insulin sensitivity is affected by factors such as age, body composition and exercise habits, which varied in our study. It is important to note that it is not clear whether increased insulin resistance would heighten or lessen our findings, although Kang et al. (2023) showed larger changes in blood glucose following exercise in diabetics. Additionally, haemodynamic changes may also help explain increased glucose uptake in our study. Hyperaemic conditions, which are observed after exercise and stretching, can augment glucose uptake into muscle cells (Draicchio et al., 2022; Kruse et al., 2016; Pellinger & Emhoff, 2022). In our study, that particular stretch was associated with the eccentric contraction from stepping down stairs as opposed to stepping up.

The current intervention falls well short of the recommended guidelines and optimal levels of exercise for glucose control. However, one main factor to consider is exercise adoption and adherence. Employing exercise options that are perceived as light and shorter in duration may promote more willingness to participate in exercise. We have previously shown that the objective intensity of stair‐stepping exercise is underestimated by participants, with moderate intensity bouts being described as very light to light intensity (Bartholomae et al., 2018). There are a wide variety of reasons people choose not to exercise, such as lack of time, perceived difficulty and financial concerns. This study examined an exercise intervention that is simple, short and very accessible to most people while also being particularly effective. Participants' scores of RPE and max HR in this trial provided data to confirm the exercise was well tolerated and perceived as light. There is a growing body of research on what has been termed as exercise ‘snacks’, consisting of short bursts of exercise typically 1–2 min in duration, which has shown positive metabolic and cardiorespiratory effects (Francois et al., 2014; Jenkins et al., 2019; Rafiei et al., 2021). The long term, cumulative effects of such short bouts can be surprisingly substantial.

This study had several limitations. One is the demographic that was included. Healthy participants were included in this study, which can impact the glucose response observed as they are more insulin sensitive and thus have higher glucose tolerance. It is uncertain how the current intervention would affect a population of people with glucose dysregulation, such as diabetics. Kang et al. (2023) showed exercise being effective for reducing postprandial glycaemia with similar reductions in AUC for both people with and people without diabetes. However, for mean 24 h glucose levels people with diabetes experienced larger reductions than those without diabetes. Additionally, in our study, we only measured glycaemic markers at predetermined time points for 2 h following an OGTT. It is unknown whether the reduction on BG observed would continue to occur with repetition over multiple meals, days and repeated stair‐stepping exercise bouts. Another limitation is whether the reductions in BG seen in this experiment would make meaningful and clinically significant impacts on a person's overall health, specifically on long‐term measurements such as HbA1c. In people without diabetes, postprandial glucose is responsible for up to 70% of the relative contribution to HbA1c suggesting targeting these postprandial periods would be effective (Monnier & Colette, 2006). Prior research has shown that dietary modulations such as including more fibre in a meal or having a mixed meal (protein, carbohydrate and fat) can lower the acute postprandial BG response (Kim et al., 2019; Yu et al., 2014). While not undergoing an overnight fast, our subjects still abstained from food for at least 5 h before undergoing a simple glucose challenge. Whether the effects observed in this study remain robust when feedings occur closer together and/or when following a real meal in the evening and therefore translate more readily to everyday lives of people remains to be determined.

### 4.1 Conclusion

A single, 1 min bout of light‐intensity stair‐stepping decreased the change from baseline to 60 min postprandial BG levels in healthy young adults.

## AUTHOR CONTRIBUTIONS

All authors have read and approved the final version of this manuscript and agree to be accountable for all aspects of the work in ensuring that questions related to the accuracy or integrity of any part of the work are appropriately investigated and resolved. All persons designated as authors qualify for authorship, and all those who qualify for authorship are listed. Austin Morales: Modification of study design, participant recruitment, data collection, management and analysis, manuscript development, editing and correspondence. Will Wong: Participant recruitment, measurement and data collection, manuscript development. Jeff Moore: Protocol inception and modification, data analysis and manuscript development. Jochen Kressler: Protocol inception, participant recruitment, staff training and supervision, data analysis and manuscript development.

## CONFLICT OF INTEREST

The authors declare no conflicts of interest.

## FUNDING INFORMATION

None.

## Data Availability

The data that support the findings of this study are available from the corresponding author upon reasonable request.
